# Brain MRI and biological diagnosis in five Tunisians MLD patients

**DOI:** 10.1186/1746-1596-7-11

**Published:** 2012-01-28

**Authors:** Ilhem Barboura, Samir Hadded, Saber Chebel, Rachida Ben Mansour, Hinda Chahed, Mohamed-Néji Gueddiche, Mahbouba Frih-Ayed, Salima Ferchichi, Abdelhédi Miled

**Affiliations:** 1 Laboratory of Biochemistry of CHU Farhat Hached, Sousse, Tunisia; 2 Pediatric department of CHU Fattouma Bourguiba, Monastir, Tunisia; 3 Neurological department of CHU Fattouma Bourguiba, Monastir, Tunisia

**Keywords:** arylsulfatase A, urinary sulfatide, sulfatide, demylinisation, metachromatic leukodystrophy

## Abstract

Metachromatic leukodystrophy (MLD) is a recessive autosomal disease which is characterized by an accumulation of sulfatides in the central and peripheral nervous system. It is due to the enzyme deficiency of the sulfatide sulfatase i.e. arylsulfatase A (ASA). we studied 5/200 cases of MLD and clearly distinguished three clinical forms. One of them presented the juvenile form; two presented the late infantile form; and two other presented the adult form. The Magnetic Resonance Imaging (MRI) of these patients showed a diffuse, bilateral and symmetrical demyelination. The biochemical diagnosis of MLD patients evidencing the low activity of ASA and sulfatide accumulation.

Virtual slides: The virtual slide(s) for this article can be found here:

http://www.diagnosticpathology.diagnomx.eu/vs/1791578262610232

**Patients and methods:**

We studied 5/200 MLD patients addressed to us for behavioral abnormalities and progressive mental deterioration. All of them were diagnosed at first by brain MRI evidencing a bilateral demyelination, then the measurement of ASA activity using P-nitrocathecol sulfate as substrate, finally the sulfatiduria was performed using thin-layer chromatography using alpha-naphtol reagent.

**Results:**

In this study, from 200 patients presenting behavioral abnormalities and a progressive mental deterioration, we reported just 2 patients were diagnosed as late-infantile form of MLD. Only1 case presented as the juvenile form; and 2 patients with the adult-type of MLD. The brain magnetic resonance imaging (MRI) of all patients showed characteristic lesions of MLD with extensive demyelination. Biochemical investigations of these patients detected a low level of ASA activity at 0°C and 37°C; the excess of sulfatide in sulfatiduria.

**Conclusion:**

MRI is required to orient the diagnosis of MLD patients; the latter must be confirmed by the biochemical investigations which is based on the measurement of ASA activity and the excess of sulfatide showed in the sulfatiduria.

## Background

Metachromatic leukodystrophy or scholz's disease is an autosomal recessively inherited lysosomal storage disorder caused by the deficiency of arylsulfatase A (ASA). This enzyme catalyses the first degradation step of the glycosphingolipid 3-O-sulfogalactosylceramide (sulfatide). Sulfatide accumulates in white matter of the central nervous system and peripheral nerves as a result of ASA deficiency and leads to progressive demyelination and lethal neurological symptoms. Visceral organs with an excretory function such a kidney and gallbladder also store sulfatide but not so much affected functionally [1].

MLD is divided into three major clinical forms according to the age of onset. The most frequent and fatal form is the late-infantile form which starts before 4 years of age and patients die by the end of the first decade. The juvenile form of MLD includes age onset between 4 and 16 years, while symptoms of adult MLD start after puberty. In a few patients, MLD results from deficiency of the activator protein saposin B (SAP-B) [2].

For many years, since the discovery of the defect in ASA for MLD, and the easy enzymatic detection of ASA deficiency, this disease has been considered as affecting only children at the walking period, this neuropathology is characterized by a regression of previous acquisitions related to dys and demyelination [3]. More precise data on the topographical sequences of myelination in humans has been recently developed by Magnetic Resonance Imaging.

The aim of this study is to underline the value of MRI for MLD diagnosis orientation and the interest of the biochemical study that involves the measurement of ASA activity and sulfatiduria to confirm MLD diagnosis.

## Patients and methods

The patients in this study were unrelated and originated from different geographic areas of Tunisia. This study was approved by ethics committees of the respective Tunisian hospitals, and the families gave informed consent before withdrawal of blood. The studied patients were addressed to us for behavioral abnormalities and a progressive mental deterioration. The patients were always consanguineous.

Family and history description of the 5 studied patients were summarized in table 1.

**Table 1:** Description of the 5 MLD patients.

Features	**Patient F.S**.	**Patient S.C**.	**Patient Y.R**.	Patient N.M.A	**Patient K.H**.
**Consanguinity of the parents/degree**	-	1^st^degree	1^st^degree	1^st^degree	1^st^degree
**Age of diagnosis (yr)**	43 yr	34 yr	4 yr	3 yr	2 yr
**Age of onset (yr)**	42 yr	33 yr	4 yr	3 yr	2 yr
**Sex**	female	Female	Female	Male	Female
**Age (yr)**	Died at 44 yr	34 yr	5 yr	4 yr	3 yr
**ASA activity at 37°C (UV: 12-35 μkat/Kg of Proteine)**	7	6	6	10	4
**ASA activity at 0°C (UV: 3-9 μkat/Kg of Proteine)**	1	2	2	1	2
**Qualitative sulfatiduria**	Presence of an abnormal band of 3-O-sulfogalactosylceramide (sulfatide)				

yr: year, UV: usual value

### Cases with late-infantile form

#### Patient: M.A.N

**Figure 1:**
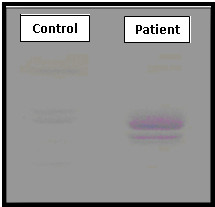
**The sulfatiduria profile of the patient compared with a control**.

**Figure 2:**
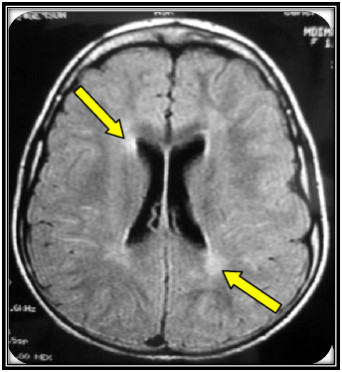
**Brain MRI of the patient with late infantile form of MLD**.

This boy was born as a child of healthy first degree consanguineous parents originated from the Sahel of Tunisia (Teboulba). The patient was delivered vaginally after an uncomplicated full-term pregnancy. He was admitted to the pediatric department of CHU Fattouma Bourguiba of Monastir at 3 years. He had difficulty in walking, nystagmus, spontaneous contraction at extremities. He had mental-motor retardation and was diagnosed as late infantile form of MLD with low ASA activity and the excess of sulfatide showed in his sulfatiduria profile. His brain MRI indicated characteristic lesions of MLD in the white matter; Figure 1 and Figure 2.

#### Patient: K.H

This girl of 2 years was referred to the pediatric department of Nabeul hospital in the northern-west region of Tunisia. She was diagnosed as late infantile MLD; she had the same symptoms as the previous case, difficulty in walking, spontaneous contraction at extremities. She also had mental-motor retardation with low ASA activity and the chromatographic profile of sulfatiduria revealed the same excess of sulfatides showed in the first case. Her brain MRI showed the same lesions demonstrated in the first patient; Figure 1 and Figure 2.

### Case with juvenile form

#### Patient: R.Y

**Figure 3:**
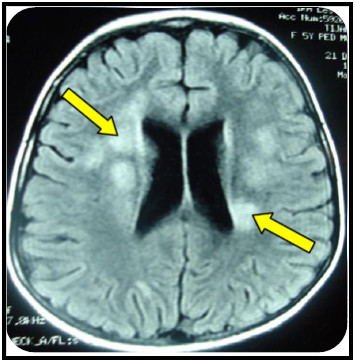
**Brain MRI of the patient with juvenile form of MLD**.

This little girl whose parents were first cousin was the second child, after a normal vaginal delivery. By the age of 5 years, this girl was evaluated for difficulty in walking at pediatric department of Sfax hospital in Southern Tunisia. After that, she lost her abilities of sitting, eating and swallowing. She had epileptic seizures, optic atrophy and spasticity. She was diagnosed as juvenile form of MLD on the basis of clinical analysis and the diagnosis was demonstrated by decreased ASA activity. Her sulfatiduria demonstrated the same results of the patients with late infantile form. Her brain MRI showed characteristic lesions of MLD; Figure 1, Figure 3.

### Cases with adult form

#### Patient: S.F

**Figure 4:**
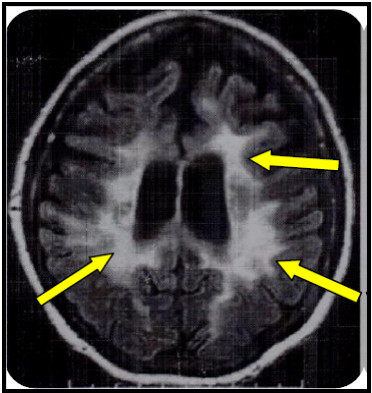
**Brain MRI of the patient with adult form of MLD 'arrows' indicate the lesions of the white matter**.

A 43-year-old female, originated from the Sahel of Tunisia (Mahdia), presented a history of primary sterility. She was admitted to the neurological service of CHU Fattouma Bourguiba of Monastir for behavioural modifications, she showed gradual obvious dementing symptoms such as memory disturbance and disorientation. We also detected some neurological signs, i.e. pyramidalor cerebellar syndrome and peripheral neuropathy. The cognitive deficits encompassed several cognitive domains including memeory, visio-spatial, and executive functions indicating widespread organic lesions, and based on the initial brain MRI, evidencing a diffuse bilateral demyelination often symmetrical, sometimes limited to the periventricular areas and respecting U fibres; Figure 4. Her ASA activity in peripheral blood leukocytes was decreased at 0°C and 37°C, the sulfatiduria profile revealed the excess of sulfatides like other cases; Figure 1. This patient died at 44 years old.

#### Patient: C.S

A 34-year-old female was referred to the neurological department of Fattouma Bourguiba Hospital of Monastir (Sahel). She presented both mental deterioration and behavioural modifications at the onset of the disease. These modifications lead her to psychiatric institutions. During evolution, other modifications appeared such as memory dysfunction, ideation difficulties, spatio-temporal disorientation, and loss of sense of judgement. The brain MRI showed the same lesions of the patient S.F; Figure 4. During evolution, the patient had increased motor and cerebellar symptoms. This lead to biological investigations, her ASA activity was low and her sulfatiduria showed the same abnormal band of sulfatide like the other patients; Figure 1.

## Methods

### ASA activity determination in leukocytes

ASA was determined in leukocytes which were isolated from blood samples of patients and homogenized by three times of freeze-thawing [4]. Proteins were determined in leukocytes homogenates according to the method of lowry using bovine serum albumin as standard. ASA activity was determined in leukocyte homogenates that contain 100-400 μg protein using p-nitrocathecol sulfate (sigma Chemical Co.,USA) as substrate [5].

The excess of sulfatides must be evidenced to confirm the diagnosis as there are pseudo-deficiencies of ASA: it was either detected on a peripheral nerve biopsy showing characteristic metachromatic deposits, or more easily looking for sulfatiduria in urine [6].

### Qualitative sulfatiduria

This method was performed as followed: a 10 ml fresh urine sample was collected; 2 drops of concentrated acetic acid were added, after that the sample was left overnight in the cold; the pellet obtained after centrifugation 10 min at 2500 rpm, was collected and extracted by 5 ml chloroform/methanol 2:1 (v/v). After sonication, 1 ml distilled water was added; after mixing throughly and centrifugation, the upper phase contained urinary salts, and the lower phase, the lipid extract. The latter was evaporated to dryness and redissolved in 100 μl of chloroform:methanol 2:1for chromatography on silica-gel thin-later plates in chloroform:methanol:water 70:30:4 by volume; the revelation of the sulfatide glycolipid was done using alphanaphtol reagent [7,8].

## Results

In This study, we estimated for the first time in Tunisia, the incidence of this neuropathology in our studied population; we founded just 5/200 cases representing the three MLD phenotypes. All suspected patients have a collapsed ASA activity in leukocyte homogenate and their chromatographic profiles were similar and revealed the presence of an abnormal band corresponding to the excess of 3-O-sulfogalactosylceramide and their brain MRI showed characteristics lesions of the three MLD forms in the white matter.

## Discussion

The MLD is a lysosomal storage disease, resulting mainly from ASA deficiency which is essential for the degradation of sulfatides. Three clinical forms have been described: the infantile form, the juvenile form and the adult form. The disparity of these phenotypes reflects the difficulty of the clinical, biological and paraclinical diagnostic. The study of heterozygous subjects by determination of enzyme activity is limited because there is an overlap between the values of ARSA activity in normal subjects and heterozygous [9]. In some clinically healthy subjects or suffering from other neurological diseases than the MLD, enzyme activity observed may be collapsed, associated with PD alleles. These alleles represent 7-15% of alleles in the general population. PD in the ARSA must be recognized to avoid misdiagnosis and for prenatal diagnosis in families which coexists one allele of MLD [10].

The prenatal diagnosis is the best way to put heterozygous couple to the disease, especially in a couple who had an affected child (index case). Currently, the identification of fetal genotype and predicting the type of the disease even in the absence of family history has become easier with the use of molecular technologies [11]. It is important to note that molecular diagnosis of MLD is more accurate and uses sample more stable than those used for determining enzyme activity. A molecular study is necessary to screen for the mutations causing different forms of this neuropathology. The characterization of these mutations allows us to clarify the genotype/phenotype correlations, to improve the quality of genetic counseling [12].

The conventional therapeutic approaches are essentially symptomatic. They were made in order to restore the arylsulfatase A activity and prevent the progression of the pathological accumulation of sulfatides and consequently reduce morbidity associated with MLD. The aim of enzymatic therapy is to administer MLD patient with active enzyme, this approach will be the future of metachromatic leukodystrophy [13].

## Conclusion

The lysosomal storage diseases, especially the sphingolipidoses, presented a great clinical variability which complicated the diagnosis. According this study, the brain magnetic resonance imaging (MRI) was required to orient the diagnosis of MLD patients. The biochemical investigations such as the measurement of ASA activity and qualitative sulfatiduria were necessary to confirm MLD diagnosis. It is important to note that according this work, we estimated for the first time in Tunisia, the incidence of this neuropathology in our studied population; we found just 5/200 cases representing the three phenotypes of MLD.

## List of abbreviations

ASA: arylsulfatase A; MLD: metachromatic leukodystrophy; MRI: magnetic resonance imaging.

## Competing interests

The authors declare that they have no competing interests.

## Authors' contributions

IB carried out the enzymatic activities and the extraction of urinary sulfatides and drafted the manuscript. SH carried out the clinical diagnosis of patients with late infantile and juvenile form of MLD. SC performed the clinical diagnosis of patients with adult form of MLD. RB.M participated in the development of the used techniques. HC participated in the preparation of reagents. MN.G participated in collecting clinical data of patients with infantile and juvenile form of MLD. MF.A participated in collecting clinical data of patients with adult form of MLD. SF conceived of the study, and participated in its design and coordination. AM also participated in the study design and coordination. All authors read and approved the final manuscript.
